# KeaA, a *Dictyostelium *kelch-domain protein that regulates the response to stress and development

**DOI:** 10.1186/1471-213X-10-79

**Published:** 2010-07-29

**Authors:** Luciana Mantzouranis, Raquel Bagattini, Glaucia M Souza

**Affiliations:** 1 Departamento de Bioquímica, Instituto de Química, Universidade de São Paulo, Av. Prof. Lineu Prestes 748, B9 S, sala 954, 05508-000, São Paulo, Brasil

## Abstract

**Background:**

The protein kinase YakA is responsible for the growth arrest and induction of developmental processes that occur upon starvation of *Dictyostelium *cells. *yakA^- ^*cells are aggregation deficient, have a faster cell cycle and are hypersensitive to oxidative and nitrosoative stress. With the aim of isolating members of the YakA pathway, suppressors of the death induced by nitrosoative stress in the *yakA^- ^*cells were identified. One of the suppressor mutations occurred in *keaA*, a gene identical to DG1106 and similar to Keap1 from mice and the Kelch protein from Drosophila, among others that contain Kelch domains.

**Results:**

A mutation in *keaA *suppresses the hypersensitivity to oxidative and nitrosoative stresses but not the faster growth phenotype of *yakA^- ^*cells. The growth profile of *keaA *deficient cells indicates that this gene is necessary for growth. *keaA *deficient cells are more resistant to nitrosoative and oxidative stress and *keaA *is necessary for the production and detection of cAMP. A morphological analysis of *keaA *deficient cells during multicellular development indicated that, although the mutant is not absolutely deficient in aggregation, cells do not efficiently participate in the process. Gene expression analysis using cDNA microarrays of wild-type and *keaA *deficient cells indicated a role for KeaA in the regulation of the cell cycle and pre-starvation responses.

**Conclusions:**

KeaA is required for cAMP signaling following stress. Our studies indicate a role for kelch proteins in the signaling that regulates the cell cycle and development in response to changes in the environmental conditions.

## Background

The social amoebae *Dictyostelium discoideum *grows as a unicellular organism feeding on soil bacteria. Upon nutrient depletion the amoebae survive by differentiating into spores in a developmental process where 10^5 ^cells aggregate and differentiate to form a 1 mm tall organism with distinct tissues. cAMP is a molecule with multiple functions during the entire life cycle, acting both extracellularly as a chemoattractant and intracellularly as a regulator of gene expression. The protein kinase YakA has been implicated in the transition from growth to development, playing a crucial role in this process. YakA is an effector of the gene expression changes that follow starvation including the down-regulation of vegetative genes, the up-regulation of the cAMP-dependent protein kinase, *pkaC*, the adenylyl cyclase *acaA*, and the cAMP receptor *carA*. During growth YakA regulates the cell cycle, and the survival to oxidative, nitrosoative and thermal stresses. YakA impinges on the cell cycle by regulating the interval between cell divisions and the growth arrest that follows stress. PKA-C is also activated by YakA in response to treatment with compounds that generate nitric oxide and by H_2_O_2 _indicating that several stress responses in *Dictyostelium *are modulated by YakA/PKA [1-3].

PKA has been extensively characterized in *Dictyostelium *and has been implicated in the regulation of both early and late gene expression, the timing of cAMP production and cell differentiation and the coordination of fruiting body morphogenesis with the terminal differentiation of spores and stalk cells [4,5]. At the onset of development, PKA-C is required for the expression of key cAMP signaling proteins such as the aggregation-stage adenylyl cyclase, ACA, and the major cAMP receptor, cAR1 [6,7].

In *S. cerevisiae *the cAMP-PKA pathway plays a central role in the responses to changes in glucose concentration and initiates the signaling process that leads to cellular growth and proliferation. Glucose binds to the Gpr1 receptor, which activates cAMP synthesis through the Gpa2 protein. Inactivation of PKA causes yeast cells to arrest proliferation and to enter into the stationary phase G0 [8-10]. Furthermore, the cAMP-PKA pathway negatively affects the H_2_O_2 _stress response [11] by a PKA-directed phosphorylation of Msn2/4 transcription factors, resulting in the inhibition of its stress-induced nuclear redistribution [12]. The cAMP-PKA pathway also has a negative effect on Yap1-dependent transcription through a mechanism that remains unrevealed. It was shown that a strain lacking the PKA regulatory subunit Bcy1 exhibits a strong inactivation of Yap1-dependent transcriptional control, although increased levels of cAMP, capable of inhibiting Msn2/4, has no apparent effect on Yap1 pathway [13-15].

In the present work we describe a new component of the YakA/PKA pathway we called KeaA. KeaA is a member of the kelch-domain superfamily of proteins [16,17] that includes more than 30 proteins found in humans, mice, *Drosophila, C. elegans, Arabidopsis*, rice, yeasts, viruses and others. The kelch domain is a motif of 44-56 amino acids commonly found repeated 5-7 times. Each motif forms a 4-stranded β-sheet tilted around a central axis as a β-propeller [18]. Its cellular functions are diverse. Members have been found that bind to actin, regulate cell morphology, modulate gene expression and mediate protein-protein interactions [17]. KeaA also presents a zinc-finger type C3HC4 domain or RING-finger. Two different variants exist of this domain, the C3HC4-type and a C3H2C3-type, which are clearly related despite the different cysteine/histidine sequence patterns. The RING-finger domain is a specialized type of Zn-finger of 40 to 60 residues that binds two atoms of zinc and is probably involved in mediating protein-protein interactions [19]. Various RING finger proteins also exhibit binding to E2 ubiquitin-conjugating enzymes [20]. Conjugation reactions related to ubiquitination are essential for macroautophagy in *Dictyostelium*. *Dictyostelium *macroautophagy mutants do not show abnormalities during growth but fail to complete normal morphogenesis. Furthermore, the development of these mutants is more aberrant in plaques on bacterial lawns than on nitrocellulose filters [21].

A mutation in KeaA has been previously described by Loomis and colleagues http://www-biology.ucsd.edu/others/dsmith/dictydb.html to affect *Dictyostelium *development. Our results are the first to indicate a role for KeaA, in the regulation of *Dictyostelium *growth and stress survival.

## Results

### *keaA*, a kelch-domain protein, suppresses *yakA*

**Figure 1:**
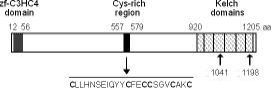
**KeaA domain distribution**. **T**he zf-C3HC4 (RING-finger) domain, the cysteine-rich region and the six Kelch repeats are indicated. The accession number in Dictybase http://dictybase.org/ is DDB_G0271500. The numbers indicate the amino acids that mark the beginning and the end of each domain, as well as the plasmid insertion sites I1041 (DG1106) and I1198 (*keaA*^- ^cells).

Treatment of cells with H_2_O_2 _or SNP (sodium nitroprusside, a generator of nitric oxide), inhibits growth of wild-type cells with little loss of cell viability but causes death of *yakA *null cells [3]. To identify components that might modulate the nitrosoative/oxidative stress responses in *Dictyostelium*, *yakA^- ^*second site suppressors were isolated from a pool of insertion mutants treated with SNP. DNA from a confirmed SNP resistant clone was isolated, the mutated gene was cloned by plasmid rescue and its sequence determined. Gene DG1106, previously mutated by the Developmental Gene Program http://www-biology.ucsd.edu/others/dsmith/dictydb.html and [Dictybase: DDB_G0271500], sequenced by the *Dictyostelium *Genome Project [22] were identical to the mutated gene. Similar proteins identified using the BLAST algorithm were the rat and mouse kelch-like protein 8 [Genbank:XP_213995], [Genbank:NP_848856], the *Drosophila *Diablo protein [Genbank:AAF43447], Keap1 [Genbank:BAA34639] and several other kelch proteins that contain a BTB/POZ domain. The gene codes for a kelch domain protein of 1207 amino acids with six kelch repeats at the C-terminus and a zinc-finger type C3HC4 domain, also called RING finger, at the N-terminus. No BTB/POZ domain is evident. An additional feature is a cysteine-rich sequence located in the mid6 portion of the protein, between the RING-finger domain and the kelch repeats (Figure 1). Due to its similarity to kelch proteins, the isolated mutant was named *keaA*^- ^and the gene *keaA*.

**Figure 2:**
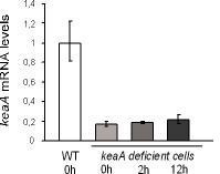
***keaA *expression in *keaA *deficient cells**. Wild-type cells and *keaA *deficient cells were diluted to 0,5 × 10^6 ^cells/mL in HL5, grown and collected when cells reached 1 × 10^6 ^cells/mL. This time point was considered the point 0 h. *keaA *deficient cells were collected at the indicated times. The ratios are relative to transcript levels detected in wild-type cells at 0 h.

The levels of mRNA for *keaA *were compared between wild-type cells and *keaA*cells by quantitative PCR. Figure 2 shows that the levels of expression of *keaA *in *keaA*cells are around 80% lower when compared to the levels in wild-type cells. For these experiments we used the REMI mutant containing the insertion of the plasmid at the end of the gene - between the amino acids 1198 and 1199. Similar results were obtained when DG1106 was analyzed (insertion at 1041).

In an effort to obtain a complete ablation of gene expression we designed a knockout vector containing 1 kb above the gene and 1 kb below the gene flanking the blasticidin resistance gene. The construct was introduced into *Dictyostelium *cells for deletion of the gene. After scanning hundreds of clones for phenotypical alterations 96 clones were evaluated for a deletion of the gene by PCR. We could not get any mutant that contained the construction recombined at the targeted site.

To confirm a role for *keaA *in the protection from stress responses the mutation was recapitulated by homologous recombination in the *yakA/keaA *null background [23] and the survival rates of *keaA *deficient cells were scored after oxidative and nitrosoative stresses. Since YakA, PKA-C and AcaA have all been shown to have a role in the ability to survive nitrosoative and oxidative stresses [3] the null mutants for these genes were also analyzed. Wild-type, *keaA^-^, yakA*^-^, *pkaC^-^, acaA^- ^*and the double *yakA^-^/keaA^- ^*cells were treated with 500 μM SNP or 500 μM H_2_O_2 _for 24 hours. The drugs were then removed and the cells plated for colony scoring after growth in the presence of bacteria. As seen in Table 1, a mutation in *keaA *rescues the hypersensitivity of *yakA*^- ^cells to both treatments. A comparison of *yakA^- ^*cells with the double mutant *yakA^-^/keaA^- ^*indicates that the double mutant shows higher survival rates. The single mutant *keaA^- ^*shows higher survival rates than those observed for wild-type cells as was also observed for *pkaC^- ^*and *acaA^- ^*cells.

**Table 1:** Survival of cells submitted to nitrosoative and oxidative challenges.

Strain	Treatment	% survival
Wild-type	SNP 24 h	86.3 +/- 4.2
*pkaC^- ^*	SNP 24 h	96.3 +/- 2.1
*acaA^- ^*	SNP 24 h	96.0 +/- 2.7
*yakA^- ^*	SNP 24 h	31.7 +/- 6.2
*keaA^- ^*	SNP 24 h	90.4 +/- 3.8
*yakA^-^/keaA^-^*	SNP 24 h	88.1 +/- 3.6
Wild-type	H_2_O_2 _24 h	70.2 +/- 4.8
*pkaC^-^*	H_2_O_2 _24 h	90.8 +/- 2.6
*acaA^-^*	H_2_O_2 _24 h	94.6 +/- 2.9
*yakA^-^*	H_2_O_2 _24 h	18.9 +/- 3.2
*keaA^-^*	H_2_O_2 _24 h	100.0+/- 4.3
*yakA^-^/keaA^-^*	H_2_O_2 _24 h	88.1 +/- 3.7

Exponentially growing cells were diluted to 1 × 10^6 ^cells/ml and incubated with 500 μM SNP or 500 μM H_2_O_2 _for 24 h. Cells were counted and plated in association with *Klebsiella aerogenes *for colony number scoring. The percentage of survival is relative to the untreated control. The results are significant of six independent experiments.

**Figure 3:**
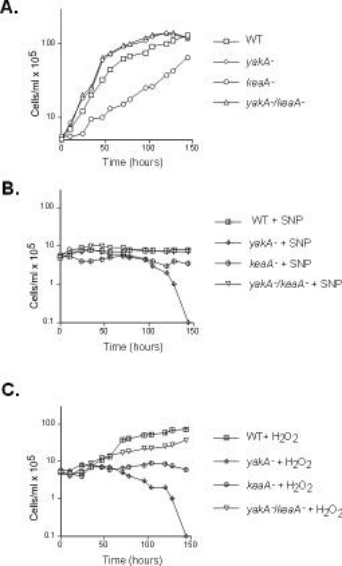
***keaA *suppresses *yakA *death induced by SNP and H_2_O_2_**. Cells were diluted to 5 × 10^5 ^cells/mL and incubated at 22°C in the absence **(A) **or presence of 500 μM SNP **(B) **or 500 μM H_2_O_2 _**(C)**. Cells were counted at the indicated times. Data corresponding to *yakA^- ^*and *yakA^-^/keaA^- ^*overlap.

*yakA^- ^*cells have been shown to have a faster cell cycle. To determine if a mutation in *keaA *might suppress this phenotype growth curves were performed. Figure 3A shows a growth curve of wild-type, *keaA^-^, yakA*^- ^and *yakA^-^/keaA^- ^*cells. The double mutant *yakA^-^/keaA^- ^*shows growth rates similar to *yakA^- ^*cells, indicating that the faster cell cycle phenotype of *yakA^- ^*is not suppressed by a mutation in *keaA*, whereas *yakA *appears as a suppressor of *keaA *null cells for growth. Also, the growth profile of *keaA *deficient cells indicates that this gene is necessary for growth. The approximate doubling time for *keaA *deficient cells during exponential growth is 20-24 h while for wild-type cells it is around 8 h. This growth deficiency is even more pronounced when cells are diluted to very low density (under 10^5^/mL). Under these conditions the doubling time rises to 40 h and frequently no growth is observed. A slower growth is also observed when cells are plated in the presence of *Klebsiella aerogenes*. Wild-type colonies take around 42 hours to appear on plates while *keaA *deficient cells take an additional 12 hours to do so.

*yakA^- ^*cells lyse under prolonged incubation in the presence of SNP and H_2_O_2_. This has been interpreted as a lack of ability to arrest growth under these conditions. To access if a mutation in *keaA *prevented such extensive damage, growth curves were performed for wild-type, *keaA^-^, yakA*^- ^and *yakA^-^/keaA^- ^*cells incubated in the presence of SNP (Figure 3B) and H_2_O_2 _(Figure 3C) for more than 5 days. As seen in the figures, a mutation in *keaA *suppresses *yakA*^- ^cell death induced by SNP and H_2_O_2_. The SNP-treated double mutant yakA-/*keaA^- ^*shows a growth profile similar to treated wild-type cells and no extensive lysis is observed. Similarly, no lysis is observed when the double mutant was treated with H_2_O_2_. The *keaA *deficient cells show a slower growth under prolonged SNP and H_2_O_2 _treatment when compared to wild-type cells. This would lead us to conclude that *keaA *minus cells are more hypersensitive to these treatments when compared to wild-type cells. But since this mutant shows impaired growth under axenic conditions in the absence of treatments, it is difficult to compare the protective role for the mutation in axenic growth. We thus decided to plate for growth in the presence of bacteria, after removal of the challenge. Under this conditions the protection is apparent since *keaA *deficient cells show higher survival rates than those observed for wild-type cells in both treatments (Table 1).

### *keaA *expression is regulated during growth and development

**Figure 4:**
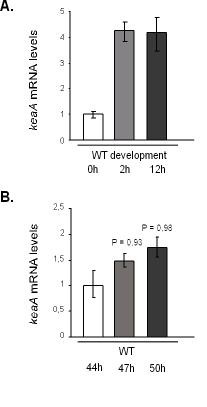
***keaA *expression is induced by growth and development**. Quantitative PCR was used to determine *keaA *expression levels during growth and development. **(A) **Wild-type cells were developed on phosphate buffer on Millipore filters and collected after 2 and 12 hours of development. The point 0 h represents wild-type cells in the early exponential phase of growth. The ratios are relative to transcript levels detected at 0 h (1 × 10^6 ^cells/mL). (**B) **Wild-type cells were grown in the presence of bacteria and collected by differential centrifugation after 44, 47 and 50 hours from plating. At 50 h plates were cleared of bacteria and development started. The ratios are relative to transcript levels detected at 44 hours. The P value was calculated in relation to the sample 44 h (reference sample). The results are representative of four different experiments.

To verify if *keaA *expression is regulated during development, wild-type cells were starved in nitrocellulose filters and the cells were collected after 2 and 12 hours. Transcript levels analysis by quantitative PCR (Figure 4A) indicates an induction upon starvation. *keaA *expression was also analyzed in wild-type cells during growth (Figure 4B). Wild-type cells were grown in the presence of bacteria and collected by differential centrifugation after 44, 47 and 50 hours. At 50 h the bacterial lawn was cleared and development started. The results indicate that an induction of *keaA *also occurs as the cells grow and the food source becomes scarce.

### KeaA is required for the production and detection of cAMP

We have shown that SNP and H_2_O_2 _treatments induce cAMP production [3]. Mutants that lack the ability to synthesize cAMP, such as *pkaC^- ^*and *acaA^- ^*cells are more resistant to oxidative and nitrosoative stresses. To determine if the observed resistance of *keaA *deficient cells to nitrosoative and oxidative stresses was related to a deficiency in cAMP synthesis, the levels of this cyclic nucleotide were measured in *keaA *deficient cells (Table 2). SNP and H_2_O_2 _treatments induce cAMP accumulation in wild-type cells but the induction is not significant in *yakA*^-^, *acaA^- ^*and *keaA *deficient cells. This indicates that these genes are necessary for cAMP production in response to these stresses.

**Table 2:** cAMP measurements of cells submitted to nitrosoative and oxidative challenges.

Strain	pmol cAMP/10^7 ^cells
Wild-type	0.95 +/- 0.15
Wild-type + SNP	2.35 +/- 0.45
Wild-type + H_2_O_2_	1.85 +/- 0.22
*acaA^-^*	1.02 +/- 0.36
*acaA^- ^*+ SNP	0.70 +/- 0.02
*acaA^- ^*+ H_2_O_2_	0.88 +/- 0.03
*yakA^- ^*	1.11 +/- 0.20
*yakA^- ^*+ SNP	1.15 +/- 0.15
*yakA^- ^*+ H_2_O_2_	1.08 +/- 0.06
*keaA^- ^*	1.01 +/- 0.08
*keaA^- ^+ *SNP	1.32 +/- 0.27
*keaA^- ^+ *H_2_O_2_	1.26 +/- 0.16

Exponentially growing cells were diluted to 1 × 10^6 ^cells/ml and incubated with 500 μM SNP for 24 h or H_2_O_2 _for 12 h. Cells were counted, aliquots of 5 × 10^6 ^cells were collected and the cell pellets were frozen for cAMP measurements. The values represent the mean +/- s.e.m. for 3 independent experiments.

To further investigate a role for *keaA *in the regulation of cAMP metabolism, wild-type and *keaA *deficient cells were starved in phosphate buffer in liquid suspension for 2 h and then stimulated with 80 nM cAMP every 6 minutes for 4 h. Table 3 shows the percentage of isolated cells counted for each cell line submitted or not to cAMP pulses. Over 80% of the wild-type cells were found in aggregates after 6 h and this number increased to 95% when cAMP pulses were added. In the case of *keaA *deficient cells, 62% were aggregated after 6 h, and addition of cAMP increased the aggregated cell population to 71%. Thus, the addition of cAMP pulses can stimulate aggregation of wild-type cells more efficiently than of *keaA *deficient cells, indicating that the mutant cells are less responsive to cAMP pulses.

**Table 3:** Stimulation of aggregation by cAMP pulses.

Strain	% of isolated cells
Wild-type	20.1 +/- 2.5
Wild-type + cAMP	5.6 +/- 1.6
*keaA^-^*	38.6 +/- 3.1
*keaA^- ^*+ cAMP	29.2 +/- 2.7

Wild-type and *keaA^- ^*cells were washed, ressuspended in phosphate buffer to 1 × 10^7 ^cells/ml and starved in liquid suspension for 2 hours. At this point 80 nM of cAMP was added every 6 minutes for 4 hours. The number of isolated cells was counted and the percentage to the total number of cells was calculated.

### KeaA is required for aggregation

**Figure 5:**
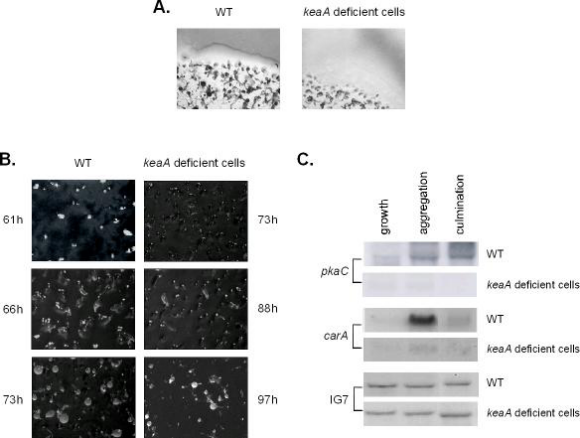
***keaA *deficient cells are aggregation deficient when grown in association with bacteria**. Development was observed for wild-type and *keaA *deficient cells grown in agar in the presence of *Klebsiella aerogenes*. **(A) **A detail of the general appearance of the colonies is shown. **(B) **The time from clearing plate formation (beginning of starvation) to aggregation, culmination and fruiting body formation is shown from top to bottom. **(C) **Wild-type and *keaA *deficient cells clearing plates were prepared and cells were collected when they were growing, aggregating and culminating. Samples of total RNA were analyzed on northern blots using fragments of *pkaC*, *carA *and IG7, a constitutively expressed gene, as probes.

A morphological analysis of *keaA *deficient cells during multicellular development indicated that, although this gene is not absolutely required for aggregation, it is required for the cells to efficiently participate in the process. Loomis described DG1106 as an aggregation minus strain after screening of colonies in agar plates. Their screening may have missed small fruiting bodies formed at a later developmental time. Figure 5A shows a detail of a wild-type and a *keaA *deficient cell colony. Wild-type cells show a defined colony border at the interface of the bacterial front, which is followed by aggregating cells, and more to the center of the colony, culminants and fruiting bodies. *keaA *deficient cell colonies are diffuse and no clear definition of the borders are seen. The region next to the bacterial front, which in wild-type cells contains aggregating cells, is devoid of aggregates. Aggregates are seen more to the center of the colonies and fruiting bodies are smaller and take longer to appear. The delay in aggregation is evident when development was observed on clearing plates. Cells were plated in the presence of *Klebsiella aerogenes *and the time to the major morphological changes during multicellular development was observed (Figure 5B). For wild-type cells ruffling was observed after 61 h from plating, culminants after 66 h and fruiting bodies after 73 h. For *keaA *deficient cells, ruffling took 12 h longer; once aggregated, the culminants took 15 h to appear and fruiting bodies another 9 h to form. Overall, the complete process took 97 h to completion in *keaA *deficient cell lines, while for wild-type cells fruiting bodies were observed after 73 h from plating with bacteria.

**Figure 6:**
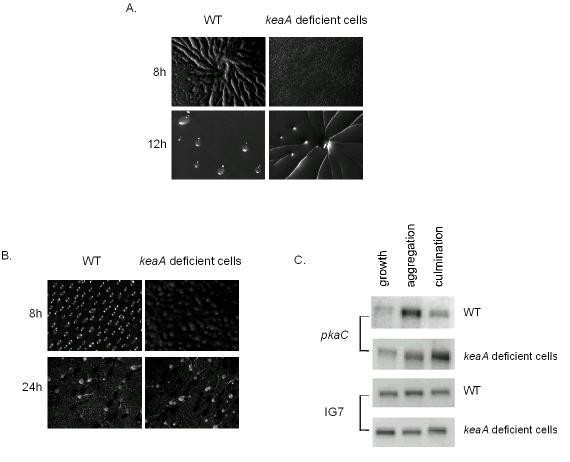
***keaA *deficient cells development is delayed when cells are starved in agar or filters**. Development was observed for wild-type and *keaA *deficient cells developed in phosphate-agar **(A) **and nitrocellulose filters **(B)**. The images were taken at the indicated time points of starvation. **(C) **Wild-type cells and *keaA *deficient cells were developed on nitrocellulose filters and collected when cells were growing, aggregating and culminating. Samples of total RNA were analyzed on northern blots using fragments of *pkaC *and IG7, a constitutively expressed gene, as probes.

The delay in aggregation appears to be dependent on the starvation conditions. When cells were starved on phosphate/agar the difference in aggregation timing was greatly diminished. Figure 6A shows the formation of wild-type spiral waves after 8 h from plating in phosphate/agar. For *keaA *deficient cells, streams of migrating cells were observed after 12 h. When plated on nitrocellulose filters the difference was smaller (Figure 6B). A delay of around 1 h is seen for *keaA *deficient cells to reach the tight aggregate stage. From then on, development seems to be very similar for both strains, with normal sized fruiting bodies being formed after 24 h.

One of the differences between the plating methods described above is the cell density at which cells were starved. For the phosphate/agar experiments, cells were plated at approximately 4.4 × 10^5 ^cells/cm^2^. For the nitrocellulose filter experiments cells were plated at approximately 31 × 10^5 ^cells/cm^2^. To test if *keaA *deficient cells were more sensitive to the density at which they were starved, the time to aggregation was measured at different plating conditions (Table 4). Wild-type and *keaA *deficient cells were plated at 1, 5, 10 and 25 × 10^6 ^cells/plate in phosphate/agar. These correspond to 0.18 cells/cm^2^, 0.9 cells/cm^2^, 1.8 cells/cm^2 ^and 4.4 cells/cm^2 ^respectively. Wild-type cells were able to form tight aggregates when cells were plated at 0.90 × 10^5 ^cells/cm^2 ^or higher. *keaA *deficient cells were not able to aggregate at this cell density. At higher densities they took longer to aggregate than wild-type cells. The time taken to complete aggregation was increasingly longer than for wild-type cells with decreasing cell densities. Taken together the results seem to indicate that the aggregation deficiency phenotype observed for *keaA *deficient cells grown in the presence of bacteria is related to the low cell density at which the cells undergo starvation under these conditions.

**Table 4:** Time to aggregation at different cell densities.

Cells/plate	Cell density (x10^5 ^cells/cm^2^)	Wild-type	*keaA*^-^
2.5 × 10^7^	4.4	9h	12 h
1.0 × 10^7^	1.8	11 h	16 h
5.0 × 10^6^	0.9	14 h	-
1.0 × 10^6^	0.18	-	

Exponentially growing cells were washed, ressuspended in phosphate buffer and plated over phosphate/agar 8, 5 cm diameter plates to the cell densities indicated on the table. The developmental process was inspected and the time to the stage of tight aggregates was determined. The dash indicates no aggregation was observed for over 36 h.

Aggregation deficiency in low cell densities was described for cells that lacked proper cAMP signaling [24]. Mutants of the adenylyl cyclase AcaA that over-expressed *pkaC *could not aggregate at low cell densities. Development of this strain was apparently normal though when the cells were plated at high cell density. To determine if components of the PKA/cAMP signaling pathway were being up regulated during aggregation as expected, the transcript levels for *pkaC *and the cAMP receptor *carA *were determined by northern blots. Wild-type cells were plated in association with bacteria and collected after 56, 61, and 66 h. *keaA *deficient cells were collected after 66 h, 73 h and 88 h. At these time points they were growing, aggregating and culminating, respectively. Figure 5C shows that *keaA *deficient cells express low levels of *pkaC *and *carA *during aggregation and culmination when plated under low cell density conditions. Presumably *carA *levels are not increased due to the lack of PKA-C accumulation.

**Figure 7:**
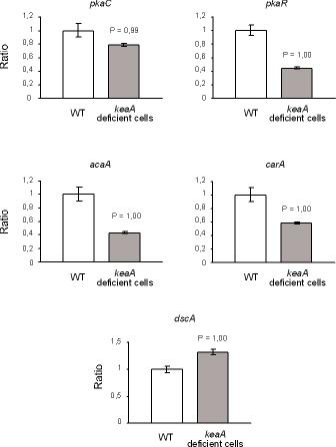
**mRNA levels of development genes in cells submitted to starvation in phosphate/agar plates**. Wild-type cells and *keaA *deficient cells were submitted to nutritional stress in phosphate/agar plates and collected after 8 h. The ratios are relative to transcript levels detected in wild-type cells at 8 h. The P value was calculated in relation to wild-type cells at 8 h (reference sample).

This may be the reason why these cells are delayed in the completion of these processes. This result was confirmed by quantitative PCR in cells developed in agar/phosphate plates (Figure 7). Moreover, keaA deficient cells express higher levels of *dscA *when compared to wild-type cells, indicating that *keaA *may be required *for *the regulation of cAMP synthesis (Figure 7). The discoidins are cytoplasmic proteins expressed several generations before the onset of starvation by the extracellular "prestarvation factor" PSF [25] and by the secreted "conditioned media factor" CMF [25,26]. Several hours after the onset of development, transcription of the discoidin genes is down-regulated by cAMP via the cell surface receptor cAR1[27]. It appears that the down-regulation of dscA is also dependent of KeaA.

When wild-type cells and *keaA *deficient cells were developed on nitrocellulose filters, the expression of *pkaC *was increased during aggregation and culmination in both cell lines but with a delay in *keaA *deficient cells (Figure 6C). Therefore *keaA *is necessary so that an increase of *pkaC *and *carA *occurs to normal levels during development especially under low-density conditions.

### cDNA Microarray

In order to define gene expression changes of *keaA *deficient cells in response to oxidative and nitrosoative stress and study in more detail the function and pathways regulated by *keaA*, hybridizations were made using cDNA microarrays. Samples from wild-type cells and *keaA *deficient cells submitted to treatments with H_2_O_2 _or SNP were compared.

**Figure 8:**
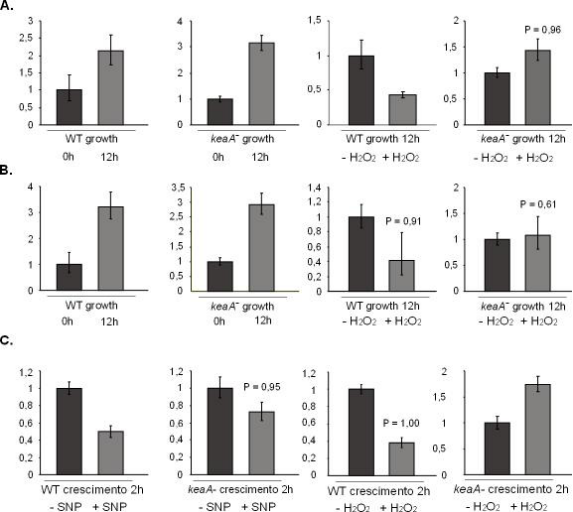
**Validation of microarray data using real-time PCR for *dscA *and *csbB***. The y axis refers to the relative expression ratio between sample versus control. In black are represented the control samples. **(A) **Discoidin A (*dscA*), **(B) **Contact site B (*csbB*). The P value represents the statistical significance and was calculated in relation to the first time point.

Data obtained using cDNA microarray was validated by qPCR. qPCR reactions were performed using samples from an independent experiment. Wild-type cells and *keaA *deficient cells were treated or not with 500 μM SNP or 500 μM H_2_O_2_. Samples of 2 × 10^7 ^cells were collected, total RNA was extracted, and cDNAs were synthesized and hybridized to cDNA microarrays or submitted to qPCR reactions. Table 5 lists the differentially expressed genes. Figure 8 shows transcript levels for a selection of genes (*dscA*, *csbB *and *cycB*) as detected by qPCR. *dscA *expression was induced in both wild-type cells and in *keaA *deficient cells. In response to 12 hours treatment with H_2_O_2 _*dscA *expression is repressed in wild-type cells but induced in *keaA *deficient cells (Figure 8A). A very similar profile was observed for *csbB *transcripts which increased during growth both in wild-type cells and in *keaA *deficient cells and decreased in wild-type cells in response to treatment with H_2_O_2 _but not in keaA deficient cells (Figure 8B).

**Table 5:** Gene expression profiling of wild-type and *keaA *deficient cells using cDNA microarrays.

	Wild type cells	*keaA *deficient cells
**0 h growth × 2 h growth**	-	-

**0 h growth × 12 h growth**	crystal protein (*cryS *) ↑	crystal protein (*cryS *) ↑
	discoidin I, A chain (*dscA *) ↑	discoidin I, A chain (*dscA *) ↑
	similar to protein kinase C inhibitor (*pkiA *) ↑	-
	12 kDa protein (*csbB *) ↑	-
	proteosomal alpha subuit 7-1 (*prtB *, M3R) ↑	-
	cytochrome c oxidase subunit VI (*cxfA *) ↓	-

**2 h - SNP × 2 h + SNP**	penta EF hand calcium binding protein (*pefA *) ↑	-
	culmination specific protein 45 D (*culD*) ↑	-
	cisteine protease 4 (*cprD*, CP4) ↓	-
	ras GTPase-activating protein ↓	-
	DG1029 (Ras GAP1) ↓	-
	G2/M - specific cyclin B (*cycB *) ↓	-
	-	putative calmodulin-binding protein CAM-BP15 (*cmbC*) ↑
	-	flavohemoglobin ↑

**12 h - SNP × 12 h + SNP**	culmination specific protein 45 D (*culD*) ↑	culmination specific protein 45 D (*culD*) ↑
	penta EF hand calcium binding protein (*pefA *) ↑	penta EF hand calcium binding protein (*pefA *) ↑
	calcium-binding protein 1 ↑	-

**2 h - H_2_O_2 _x 2 h H_2_O_2_**	glutathione reductase (*gsr *) ↑	-
	major vault protein (*mvpB *) ↑	-
	culmination specific protein 45 D (*culD*) ↑	culmination specific protein 45 D (*culD*) ↑
	-	calcium-binding protein (CBP2) ↑
	-	F-box A protein (*fbxA *) ↑
	-	hiwi ↑
	leucine-rich repeat-containing protein LRR (*lrrA *) ↓	leucine-rich repeat-containing protein LRR (*lrrA *) ↓
	unknow (*smlA *) ↓	-
	cisteine protease 4 (*cprD*, CP4) ↓	-
	putative CMF receptor - CMFR1 (*cmfB *) ↓	putative CMF receptor - CMFR1 (*cmfB *) ↓
	major vault protein (*mvpA *) ↓	-
	aquaporin like protein (*wacA *) ↓	-
	G2/M - specific cyclin B (*cycB *) ↓	-
	-	unknown (DG1008, *sfbA *) ↓
	-	sulfite reductase (*redA *) ↓

**12 h - H_2_O_2 _x 12 h H_2_O_2_**	discoidin I, A chain (*dscA *) ↓	-
	12 kDa protein (*csbB *) ↓	-

Genes with at least 60% of replicate points outside the cutoff limits in both biological samples were considered differentially expressed. The induced genes are represented by ↑ and the repressed genes by ↓.

**Figure 9:**
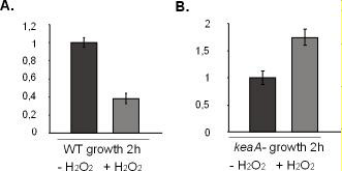
**Validation of microarray data by real-time PCR for *cycB***. The y axis refers to the relative expression ratio between sample versus control. The control samples are represented in black. **(A) **WT, **(B) ***keaA *deficient cells.

*cycB *expression was suppressed in response to treatment with SNP both in wild-type cells and in *keaA *deficient cells, but the decrease was more pronounced in wild-type cells than in *keaA *deficient cells. Treatment with H_2_O_2 _for 2 hours led to the repression of *cycB *in wild-type cells but in *keaA *deficient cells we observed increased levels of this gene's transcripts (Figure 9A and 9B).

## Discussion

**Figure 10:**
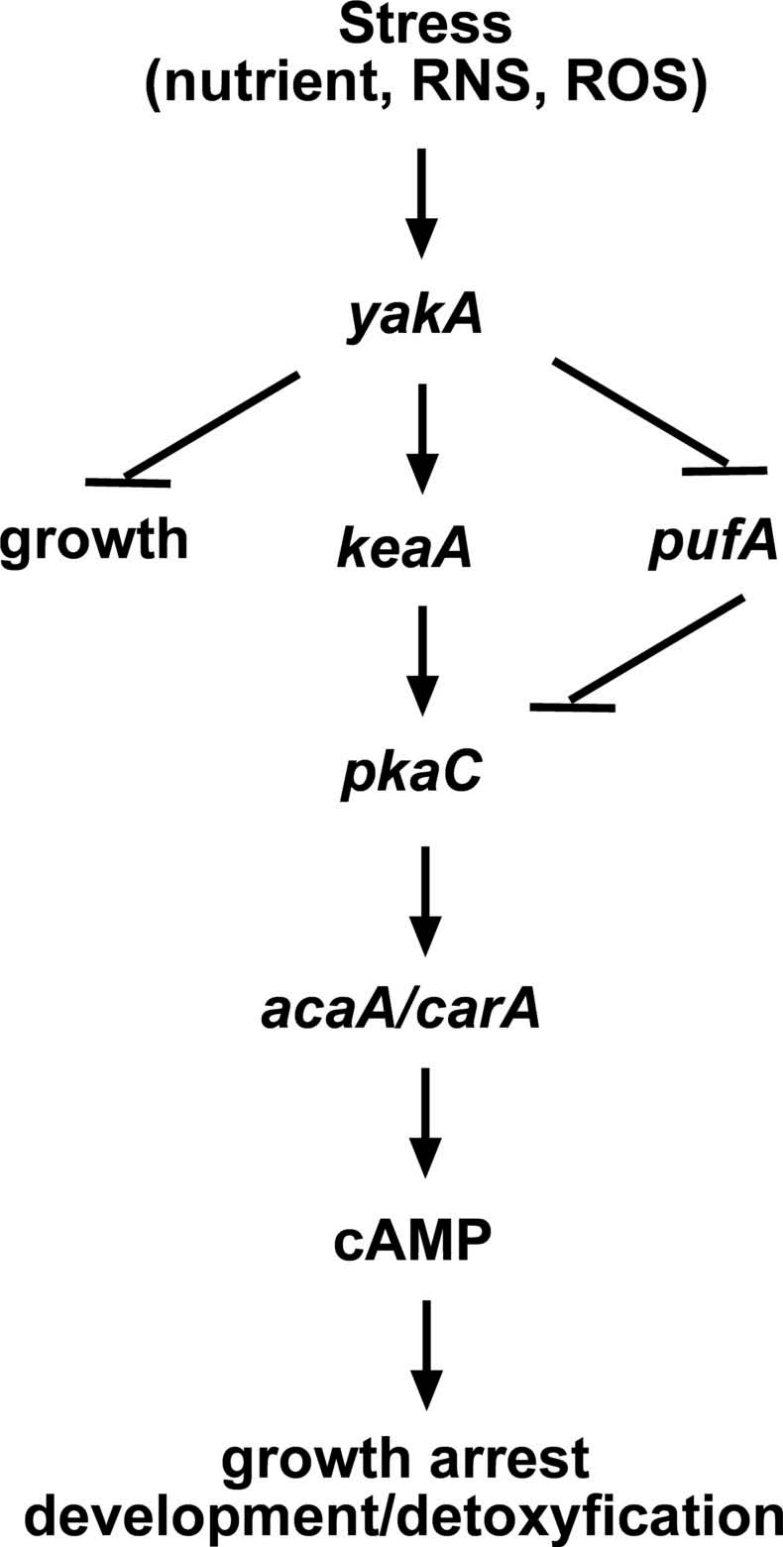
**Pathways proposed to mediate *Dictyostelium *stress responses**. The regulatory relationship between genes and events is described, with arrows representing a positive requirement for a gene or event and bars representing an inhibitory role.

We have previously shown that stimulation of cells with H_2_O_2_, SNP or spermine NONOate, a generator of nitric oxide, inhibited growth of wild-type cells [3]. The same treatment led to extensive cell lysis and death of *yakA *null cells. *pkaC *and *keaA *were isolated as suppressors of *yakA*, in a screen targeted to reveal genes involved in the survival to nitrosoative stress [3]. Both genes are regulators of the starvation response. It appears that cAMP synthesis through the adenylyl cyclase AcaA is required for nutrient and nitrosoative/oxidative stress responses and that KeaA plays a role in these processes. A model that fits the findings of this and previous work is shown in Figure 10. *keaA *is necessary for cAMP synthesis in response to prolonged nitrosoative/oxidative stress (Table 2) and also for the increased mRNA levels of *pkaC, acaA *and *carA *found during development in low cell density (Figure 5, 6 and 7). The model includes the inhibition of *pufA *expression by *yakA *in response to starvation [2]. PufA seems to also have a role in the nitrosoative/oxidative stress response since in its absence the cells are more sensitive to these stresses [3]. The model also indicates *yakA*'s regulation of the cell cycle through a pathway that is partially independent on *pufA/pkaC/keaA *to account for the fact that *yakA *null cells have a faster cell cycle which is not suppressed by second-site mutations in these genes. Furthermore, *pkaC^-^*(act6::*yakA*) and *aca^- ^*(act6::*yakA*) do not grow in liquid medium, showing that the arrest of growth is independent of *pkaC *and *acaA *[1]. This may also explain the hypersensitivity observed for the *yakA *mutant to stress conditions that require growth arrest.

Our data implicates KeaA in the regulation of cAMP metabolism during development, but only when cells are starved in low cell density. *keaA *is not required for fruiting body formation when cells are allowed to aggregate at high density. It may be that the aggregation phenotype is not so severe in *keaA *deficient cells due to the remaining 20% of expression of the truncated transcript that may yield some level of KeaA protein activity.

It is possible that KeaA acts as an adapter on the formation of a complex involved in cAMP synthesis. Kelch domains have been implicated in protein-protein interactions and also in actin-cytoskeleton interactions [17]. In its absence, the signaling might still occur but with a less than optimal efficiency which might impair chemotaxis in low cell densities probably by affecting the establishment of polarity since this requires chemoattractant stimulation, a prerequisite for migration [28]. Further studies are necessary to determine if KeaA is required for cell polarization.

A mechanism for the activation of PKA by a Gα protein named Gpa2 was proposed in yeast. The activation appears to be accomplished trough the inhibition of two repeat proteins, Krh1 and Krh2, bypassing the direct regulation exerted by adenylate cyclase [29]. Gpa2 regulates PKA activity via two distinct pathways: through stimulation of adenylate cyclase [30,31], and through inhibition of the Krh proteins. Krh1 directly interacts with PKA by means of the catalytic subunits, and Krh1/2 stimulate the association between the catalytic and regulatory subunits *in vivo *[29]. Krh1/2 also enhance the association between mouse R and C subunits, suggesting a possible conservation of Krh control of PKA [29]. We have conducted tests using the two-hybrid system to verify the interaction between PKA-C, PKA-R and KeaA but no interaction was observed (not shown).

On the other side the accumulation of intracellular cGMP and the transient polymerization of F-actin induced by folic acid stimulation has been shown to be absent in *yakA*^- ^cells [32]. The coupling of G-proteins to the cAMP receptor CarA did not seem to be affected by YakA, implicating that YakA may act downstream from CarA in the cAMP signaling pathway. KeaA may modulate this event. Since Kelch proteins have been seen to interact with actin in several other systems, it is possible that KeaA localizes to the membrane and directly interacts with the cAMP synthesis machinery, acting as an integral component of the spatially localized signaling that occurs at the cell cortex [33]. An investigation on the sub-cellular distribution of KeaA may help the biochemical characterization of its roles within the cell.

Kelch domain proteins participate in a wide variety of cellular processes. Originally they were identified in *Drosophila *associated with actin as a component of ring canals [16]. Kelch domains have been found in combination with a BTB/POZ or a Coiled/coil [17]. The association of a zf-C3HC4 domain (RING domain) with kelch domains, as seen in KeaA, is not common. Most of the kelch proteins found in metazoans are of the BTB/POZ variety [34]. RING domains appear to mediate protein-protein interactions or the assembly of multi-protein complexes [19]. 5P24, a protein with a similar architecture (RING + Kelch domains) found in *C. elegans *[Genbank:NP 506602] has been found in large-scale RNAi experiments to be essential for male fertility [35]. Is interesting to note that the *C. elegans *Puf proteins FBF-1 and FBF-2 regulate germline sex determination [36] and germline stem cell maintenance [37]. As mentioned above, in *Dictyostelium*, the Puf protein PufA, was found as a suppressor of *yakA*^-^s defects in development [2]. It is plausible that some of the same players responsible for decisions that lead to growth arrest and differentiation in *Dictyostelium *(*yakA^-^pufA-keaA-pkaC*) are at work in *C. elegans*.

A role for a kelch protein in the response to oxidative stresses has also been found in mice. The interaction of Keap1, Nrf2 and Cullin-3 forms an ubiquitin ligase substrate adaptor. Mutation of Keap1 leads to constitutive activation of Nrf2, upregulation of antioxidant genes, and resistance to electrophiles and oxidative stress [38]. On exposure to oxidative stress the sulfhydryl groups of Keap1 act as sensors. Modification of the cysteine thiols causes disruption of the Nrf2/Keap1 complex and migration of Nrf2 to the nucleus, where it induces expression of detoxifying and antioxidant genes [39,40]. Murine, rat and human Keap1 contains 25 reactive cysteines. The most reactive residues have been identified in the intervening region between the BTB/POZ domain and the Kelch domains. Experiments that address the functional role of these cysteines are under way that might reveal a link between the redox state of the cell in the transition from growth to development and a possible role for ROS and NRS in the triggering of development.

The observation that *dscA *is repressed in response to treatment with H_2_O_2 _may be related to the fact that this treatment inhibits cell proliferation as does starvation. *dscA *expression is modulated by pre-starvation [41]. YakA has also been found to be involved in the regulation of the pre-starvation response [1]. *dscA *decrease may be controlled by cAMP and since *keaA *deficient cells do not accumulate cAMP in response to treatments with SNP and H_2_O_2_, the levels of *dscA *are kept high in *keaA *deficient cells treated with these compounds. Another gene that showed a similar expression profile was *csbB*, a gene that as discoidin is involved in cell adhesion. Cell cycle may also be de-regulated in the mutant, which may explain why *keaA *deficient cells do not stop growth in response to the stresses. In wild-type cells Cyclin B mRNA decreases while in *keaA *deficient cells the decrease is less prominent in response to SNP and actually increases in response to H_2_O_2_.

## Conclusions

Our results indicate a role for KeaA in the regulation of *Dictyostelium *growth, development and stress survival. KeaA is required for cAMP signaling and the regulation of gene expression associated with the aggregation process. KeaA is also required for normal growth and oxidative/nitrosoative stress responses.

## Methods

### Cell strains

All strains are derived from the axenic *Dictyostelium discoideum *strain AX4 [42]. Mutant strains used were as follows: *pkaC *null [43], *acaA *null [44], *yakA *null AK800 [1]. The original *yakA/keaA *null strain isolated in the suppressor screen described by [3] was named 13-8. Regions flanking the plasmid insertion site in the REMI-mutant were isolated by plasmid rescue and the plasmid was named p13-8. The *yakA/keaA *recapitulated null strain GMS121 was obtained by homologous recombination using plasmid p13-8. The same plasmid was used to disrupt *keaA *in the wild-type background (strain GMS122). The insertion is located between amino acids 1198 and 1199 of [Dictybase: DDB_G0271500]. The DG1106 isolate insertion was between amino acids 1041 and 1042 http://www-biology.ucsd.edu/others/dsmith/dictydb.html.

### Growth, development and stress conditions for *Dictyostelium *cells

All strains were grown in axenic media (HL-5) or on SM agar plates in the presence of *Klebsiella aerogenes *[45]. Treatments for survival rate scoring and growth curves were performed in fresh axenic cultures kept exponentially growing in HL-5 for one week. For both cases cells were collected at 1-2 × 10^6^/mL, diluted to 0.5-1 × 10^6^/mL in HL-5 and 500 μM H_2_O_2 _or 500 μM sodium nitroprusside (SNP) were added. Cells were counted with the aid of a hemocytometer. Growth curves for mutants were determined in side-by-side tests with non-mutant sibling transformants. Survival rates were determined by counting the cells after the treatments, plating in association with *Klebsiella aerogenes *and counting the colonies formed.

For preparation of clearing plates, 3 × 10^5 ^cells were plated with 0.4 mL of an overnight culture of bacteria over SM plates. For development on agar plates, cells were washed twice in 20 mM potassium phosphate buffer pH 6.4 and plated on 1% agar dissolved in the same buffer. Cells were developed on nitrocellulose filters as described [45]. For cAMP pulsing experiments, cells were washed in 20 mM KPO_4 _pH 6.4, ressuspended to 1 × 10^6 ^cells/mL in the same buffer and shaken at 100 rpm for 2 hours, followed by pulses of cAMP to 80 nM at 6-minute intervals.

### Transformation

REMI mutagenesis was carried out in the *yakA *null background (strain AK800) as described [3]. Confirmation that a mutation in *keaA *was responsible for the resistance to SNP treatment observed in the 13-8 strain was done by recapitulation of the resistance phenotype by disruption of the gene in the *yakA *null background. Homologous recombination was carried out by electroporation of *yakA *null and wild-type cells with 40 μg of p13-8 digested with ClaI. Transformants were selected in HL-5 supplemented with 4 μg/mL Blasticidin.

### Isolation of suppressors

The screen for mutations that suppress the *yakA*-null sensitivity to SNP was carried out as described [3]. The YakA-null mutant AK800, which harbors a plasmid insertion (IS800) in the sequence that encodes the protein kinase core [1], was used as the parental strain for insertional mutagenesis. A REMI-mutagenized population of 70,000 clones was diluted to 5 × 10^5 ^cells/mL in HL-5 supplemented with 500 μM SNP. The cells were shaken at 22°C for 10 days, after which time cells were diluted and plated in association with *Klebsiella aerogenes *for clone isolation.

### DNA and RNA manipulations

Standard DNA and RNA manipulations were carried out as described [46]. Genomic DNA from the suppressor mutant was extracted and flanking genomic DNA was recovered from strain 13-8 by plasmid rescue with *ClaI *to liberate a 4.9 kb fragment which was cloned as described [47]. Plasmid p13-8 was sequenced and the insertion mapped to the open reading frame of gene DG1106 (accession number AF111942). Recapitulation by homologous recombination at the *keaA *locus using plasmid p13-8 was confirmed by digestion of genomic DNA from candidate clones with EcoRI and hybridization with a ClaI fragment probe on Southern blots. RNA was extracted using the Trizol reagent as described by the manufacturer (Life Technologies). The DNA fragments used as probes on northern blots were as follows: an EcoRI fragment containing the full length cDNA for *carA*, a BamHI/HindIII fragment of *pkaC *that excludes the repeats at the N-terminus of the protein.

### Biochemical analysis

cAMP measurements were carried out using the BIOTRAK cAMP ^125^I Assay System (dual range) (Amersham Pharmacia Biotech). Samples were prepared from cells diluted to 1 × 10^6^/mL in HL-5 media with or without SNP or H_2_O_2_. After treatment, 5 × 10^6 ^cells were spun down, ressuspended in 100 μL of phosphate buffer, added to 100 μL of 3.5% perchloric acid and frozen. Before analysis frozen samples were thawed and neutralized with 50% NaHCO_3_. The resulting lysates were centrifuged and the supernatants assayed.

### DNA and protein sequence analyses

Clone 13-8 sequence was compared to the sequences present in the databanks using the BLAST search program [48] and indicated complete identity to the DG1106 amino acid and nucleotide sequences deposited in Genbank under the accession number AF111942.

### cDNA microarrays

Probes were amplifyed by PCR directly from bacterial clones in culture. Microarrays were constructed by arraying cDNA fragments on metal-coated glass slides (Type 7, Amersham Biosciences). Each cDNA fragment was spotted on the slides at least twelve times (i.e., technical replicates). The arrays contain 237 genes represented. Following printing, the slides were allowed to dry and spotted DNA was bound to the slides by UV cross-linking. RNA extracted from wild-type and *keaA *deficient cells treated or not with SNP or H_2_O_2_. Samples from two independent experiments (i.e., biological samples) were used for the synthesis of fluorescent targets with Cy3 or Cy5.

### Data analysis of cDNA microarrays

Slides were scanned, images were processed and data collected using the ArrayVision (Imaging Research, Inc.) software. The data were extracted and normalized by Lowess fitting [49]. We used homotypic hibridizations of the reference sample to define intensity-dependent cutoff levels that would indicate differentially expressed genes as described [50]. Genes with at least 60% of the replicate points above or below the cutoff limits were considered differentially expressed in that particular sample [50].

### Quantitative PCR

RNA was treated with DNAse and used for transcription reverse reactions. The quantitative PCR reactions were carried out using the SYBR Green PCR Master Mix (Applied Biosystems) in a GeneAmp 5700 Sequence Detection System (Applied Biosystems). qRT-PCR assays were performed with the following gene-specific primer pairs: *keaA*: TTGAAACTTGGGAATGGGAAA and CAGCTAAAACCTCAGAAAAGTAACCA, IG7: GTGGTTCGGCACCTCGAT and CACCCCAACCCTTGGAAA, *dscA*: GGTGCTGCTGTTACTGGTGT and GGTGGATAGCAATTGAACGA, *pkaC*: TTTGGCACCTGAAATCATTC and AAGGTGGATAACCTGCCAAC, *pkaR*: GGT GAG GTT ATT GTG CGT CAA G and GTG ACA ACA ACT TTA CCT TCA ACG A, *acaA *: TCCTTTGGTTGCTGGTTGTA and TCTTGAGCATTGGATTGCAT, *carA*: ATGTTTCCACCAGCACTCAA and AAATGTGACAGATGCCCAAA, *csbB*: TGAAGATGGGGAATCAACAA and GGAAATATTTGGGGAGCTGGT, *cycB*: GTCCACAAATCAAGGATTTCGTT and ATTTGTCTTTCCATGTCGATAACCT. The specificity of the amplified products was evaluated through the analysis of the dissociation curves generated by the equipment. The data and error bars were calculated as described by Livak and Schmittgen [51]. To access the statistical significance of expression ratios, we assumed a log-normal model and calculated the probability P = Pr(sample > reference) and P = Pr(sample < reference). We consider expression levels to differ the reference sample on each experiment when P ≥ 0.95 [50,52].

## List of abbreviations used

SNP: sodium nitroprusside; RNS: reactive nitrogen species; ROS: reactive oxygen species.

## Authors' contributions

LM and RB carried out most of the experimental work. GMS coordinated the study, participated in its design and wrote the manuscript. All authors read and approved the final manuscript.
